# Private Media, Public Funds, and Democratic Allocation: Outlining the Civic Strategy in Media Policy

**DOI:** 10.1007/s13347-026-01035-2

**Published:** 2026-02-26

**Authors:** Rubén Marciel

**Affiliations:** Department of Political Science and International Relations, University of Geneva, Bd. du Pont-d’Arve 40, Geneva, 1205 Switzerland

**Keywords:** News media, Media policy, Democracy, Journalism, Philanthropy

## Abstract

After the digital revolution, quality journalism is more necessary than ever. And yet, journalism is undergoing an unprecedented economic crisis precisely because the digital revolution led its advertisement-based funding system to collapse. How can we save journalism as a key institution for upholding the quality of democratic deliberation? Most approaches to this question focus either on media literacy campaigns, restricting the dissemination of disinformation, or fostering public media. This article explores another, largely neglected approach: the civic strategy in media policy, which consists of distributing public funds among private news media through democratic allocation mechanisms. Civic media policies promise to create a more democratic media system, enhance media pluralism, and, crucially, secure robust funding for quality journalism. To examine how they might do so, I assess the four main proposals within this civic paradigm: Cagé’s proposal to create ‘nonprofit media organizations’, Latham’s ‘voter-funded media’ system, Ackerman’s ‘internet news voucher’, and McChesney and Nichols’ ‘citizenship news voucher’. Drawing on a critical examination of these proposals, I extract some lessons for designing more effective civic media policies in the future.

## Introduction

The digital revolution was revolutionary because, by making it extremely easy for anyone to access the means to create and share information, it democratized the capacity to communicate with the public, which until the early 21 st century was monopolized by a few. By lowering the barriers to entry into public debate, the digital revolution allowed traditionally marginalized voices to make their way into the conversation, weakened the old gatekeepers’ power to set the agenda, and, overall, created a more egalitarian public sphere. And yet, despite its immense potential to reinvigorate democracy, the digital revolution unleashed two other processes that, together, put democracy at risk (cf. Hasen, 2017). One is the so-called “information disorder” (Wardle & Derakhshan, 2017). The universalization of the capacity to produce and share content led to an explosion in the amount of information available, a non-negligible part of which is mis- and disinformation. The problem of information disorder is not, however, that there is a vast amount of information, nor even that some—or much—of that information is false. The main problem is that, in a world in which so many sources create and disseminate contents at a vertiginous pace, it is extremely costly and difficult, if not impossible, for ordinary citizens to discriminate between the contents that are truthful and newsworthy, and those that are not. 

One might think that journalists could help citizens make sense of this information disorder. Indeed, they *should* (Marciel, 2023: 376). Unfortunately, another troubling process triggered by the digital revolution is precisely the economic crisis of journalism, which has left the profession hanging by a thread. For roughly the past two centuries, the institutions that supplied most of the news sustaining democracy were commercial news media which, for better or worse, were able to inform the public thanks to their control of the bottleneck between advertisers and audiences. To promote their products, advertisers had to pay large sums to these media, which used part of those funds to produce quality journalism. However, the digital revolution dissolved that bottleneck and, as new sources proliferated, traditional journalistic outlets lost much of their advertising revenue, which began to flow to new, more profitable—but not necessarily informative—formats, such as social media and search engines. As a result, many journalistic organizations, including also many created after the digital revolution, had to shut down, in what some have referred as a “media apocalypse” (Thompson, 2017). The remaining ones had to drastically cut production costs, which in most cases entailed laying off a large portion of their staff and, consequently, offering less and lower-quality journalism (Pickard, 2019; McChesney, 2012, 2016; Cagé, 2021).

These two trends have sparked a growing debate about how to safeguard independent, high-quality journalism. Part of this debate focuses on the *demand side* of the news market, aiming to enhance citizens’ ability to identify trustworthy content and sources—for example, through media literacy (Altay et al., 2024) or the provision of metainformation (Herzog, 2023; Marciel & Magaña, 2023: 509). Other contributions concentrate on the *supply side*, seeking to ensure that citizens have access to a regular and adequate flow of quality information. On this side, there are two main approaches. The first is the *restrictive strategy*, which focuses on developing policies against disinformation (see, e.g., Brown, 2021). Along these lines, the EU has recently enacted the *Digital Services Act*, which, among other things, obligates online platforms to remove misinformative content, and which has led most EU countries to develop comprehensive policies to tackle misinformation (see Bleyer-Simon et al., 2024: 162–163). The second approach is the *public media strategy*, which aspires to ensure the existence of a minimum supply of quality news by creating and funding publicly owned media, such as the BBC in the UK or RTVE in Spain. The public media strategy rests on the assumption, well supported by empirical evidence, that well-funded and independent public media tend to produce high-quality journalism (see, e.g., Aalberg et al., 2010; Benson et al., 2017).

This article explores another, largely overlooked approach, which I refer to as the *civic strategy in media policy*, and which could serve as a complement to the existing ones. In essence, the civic strategy aims to support civil society in creating and sustaining high-quality journalism by enabling citizens themselves to distribute public funds among different private outlets. It thus combines three key components: private media, public funding, and democratic allocation.

Of course, many democracies have allocated public money to private media. The European countries, especially the northern ones, have a strong tradition of publicly subsidizing private news media. To a lesser extent, the United States has also funded private news media with public funds, for instance by heavily subsidizing newspaper delivery through the postal service during the 18th and 19th centuries (McChesney & Nichols, 2010; Pickard, 2019). However, the possibility that citizens themselves distribute public funds among media outlets of their choice has so far received very limited attention: in most cases the public money is directly distributed by officials or according to predetermined rules—such as those automatically awarding funds to the second- or third- largest newspaper in a region (see Ots & Picard, 2018).

It is true, though, that some countries do allow citizens to spend public money on news media. In Hungary, for instance, since 1996 the so-called “1% law” has enabled citizens to transfer 1% of their personal income tax to NGOs of their choice, which may also include news media (Starkman & Chittum, 2021: 244–248). More recently, France and Spain have introduced cultural vouchers granting a fixed amount of money to teenagers, to be spent on cultural goods—including subscriptions to news media. These schemes, however, are not specifically designed to fund journalism, but more generally to support public goods, among which journalism might be, if anything, only one. They thus offer no guarantee that quality journalism will be actually funded. The proof is that, even though in Hungary the “1% law” is widely used to fund news media organizations, in Spain most of the cultural voucher euros go to music festivals and shopping malls (Jara & Sánchez, 2024). In recent years, however, several scholars have advanced proposals that would enable citizens to decide how public funds specifically aimed at funding journalism should be distributed (Ackerman, 2011; Cagé, 2016; Hind, 2010; McChesney & Nichols, 2010; Latham, 2012). These proposals—and, more generally, civic media policies—are promising on at least three fronts (cf. McChesney & Nichols, 2010: 205–206; Latham, 2007: 25ff). First, and most obviously, they seem more directly democratic, since the allocation of public funds would not be discretionarily decided by government officials, decided automatically through abstract rules, nor conditioned by wealth imbalances—as it typically happens in market- (Baker, 2001: pt. 1) and philanthropy-funded media (Benson, 2018; Usher, 2021: chp. 7). Second, they promise to increase media pluralism: letting ordinary citizens decide how funds are distributed, rather than relying on public and bureaucratic mechanisms, could better harness the innovative potential of civil society. Third, civic media policies promise to foster the proliferation of independent, well-funded news organizations capable of producing high-quality journalism. The reason for this expectation is that civic media—as I refer to the organizations funded through such policies—would be hybrid institutions, somehow in between traditional private and public media, which could allow them to combine the most valuable traits of both. Being privately owned and privately run, civic media could maintain a strong independence from the state, as it’s typical of private outlets. At the same time, being publicly funded, they could be invulnerable to both market pressures and the influence of wealthy donors, as is typically the case with public media.

Of course, the devil lies in the details, and whether civic media policies can deliver on these promises largely depends on how specifically they are designed.^1^ My aim in this article is precisely to play the devil’s advocate by critically assessing the four most compelling proposals within the civic media paradigm, to identify their main shortfalls. Admittedly, this is a rather negative endeavor. I hasten to note, however, that my critical approach is not meant to demerit these proposals, which I find groundbreaking and inspiring. Indeed, I focus on them precisely because developing the civic media strategy requires examining its most promising proposals, so that we can envision *even better* ones. Despite its harsh appearance and seemingly destructive purpose, my criticism is thus sympathetic and constructive, for it ultimately aims to learn from those who put more thought on the civic strategy, in order to pave the ground for its further development.

Before proceeding, let me briefly explain the two desiderata I will use for my assessment. The first is *quality journalism*, which stands for the aspiration that civic media policies support only news organizations well-equipped to produce quality journalism. Although there are different accounts of quality journalism (Christians et al., 2009), here I assume the *deliberative ideal*. In this view, the main function of journalism is to promote high-quality democratic deliberations by providing citizens with the information needed to engage in such deliberations (Ettema, 2007; Marciel, 2025). To fulfil this function, journalistic outlets should be able to guide their editorial decisions by the standards of public reason (Ettema, 2007; Fox, 2013). This in turn requires them to be not just minimally *professional*, but also minimally *inclusive and representative* of the population they serve (Girard, 2015; cf. Bleyer-Simon et al., 2024: 6; Kurtulmuç and Kandiyali, 2023), and strongly independent from political, economic, and social pressures that might compromise their editorial autonomy and deviate them from their public mission (Bleyer-Simon et al., 2024: 112; Maia, 2018: 351–353).

The second desideratum is *media pluralism*, understood as the aspiration that civic media policies foster a diverse civic media subsector. To be clear, I do not assume that the entire media system should abide by the standards that apply to media whose role is to produce quality journalism. As McChesney and Nichols (2010: 164) note, what matters is that “the media system as a whole makes such [quality] journalism a realistic expectation”. Accordingly, I assume that a healthy media system can be conceptually divided into two sectors (cf. Curran, 2002: 239–247; Picard & Pickard, 2017). One is the journalistic sector, composed of media organizations whose main function is to provide citizens with the news they need to be well informed and able to make well-grounded political choices. The other is the non-journalistic sector, composed of media institutions pursuing other legitimate objectives besides promoting a well-informed citizenry—such as entertaining, lobbying, or advancing a certain religious faith. Although some standards of media pluralism might be applicable to this non-journalistic sector, in this paper I am concerned only with the journalistic one.

As conceived here, the standard of media pluralism entails the assumption that journalism’s main function is best served when the journalistic sector incorporates a plurality of news media—not only with different editorial lines, but also with diverse ownership and organizational structures. Indeed, some of the media that could—and, perhaps, *should*—constitute this journalistic sector are what I refer to as *civic media*, that is, private media that are (at least partially) funded with public funds directly distributed by citizens. If these media existed, they would constitute a civic subsector within the journalistic sector. When applied to this civic media subsector, the desideratum of media pluralism entails three distinct aspirations: first, n*umerical pluralism*: there should be a sufficient number of civic media; second, *viewpoint pluralism*: the civic media subsector should reflect a wide variety perspectives; and, third, *format pluralism*: the civic media subsector should include civic media of different kinds (e.g., daily newspapers, weekly podcasts, TV news programs).^2^

## Cagé’s Nonprofit Media Organizations

The first proposal is owed to Cagé (2016: Chap. 3; see also Cagé, 2021). Cagé’s idea is to enable the creation of a new type of association she calls *Nonprofit Media Organizations* (hereinafter NMO).

NMOs would exhibit three key characteristics, combining features of foundations and joint-stock companies. First, like foundations, funds invested in NMOs would be irrevocable: once donated, shareholders could not recover their investment.^3^ Notably, NMOs could also accept other sources of funding, including advertising. Second, NMOs would be nonprofit entities, which means that their profits should be reinvested in their activities rather than distributed. Third, akin to joint-stock companies, donations would confer participation rights, but not in a strictly proportional way: large investors would receive voting rights at a sub-proportional rate—proposed to be one-third after 10% of capital—while small investors would get voting rights at more than a proportional rate.

The purpose of such disproportionality is to promote democracy within NMOs. Since such a voting scheme might disincentivize rich donors, Cagé suggests compensating donors through tax incentives: as with foundations, donations to NMOs would be tax-deductible. Note that this amounts to an *indirect* public subsidy: through tax incentives the state abstains from collecting tax money, which remains in the hands of those exempted from taxation. This indirect, yet *public* funding is what makes Cagé’s proposal a civic one—albeit a borderline case. And this *indirect* character of the public funding is, as I argue now, the Achilles’ heel of her proposal.

To begin with, Cagé’s bylaw would legalize a new type of association (NMOs), but since it grants no funding to promote their creation, it offers no guarantee that such associations will actually emerge nor that, if created, they will be enough in number. Their existence would thus entirely depend on individuals voluntarily investing their resources in NMOs—that is, on *charity*. This is problematic because today most citizens are currently unwilling to pay for journalism (Goyanes et al., 2022)—which partially explains why the non-profit journalistic subsector is still small and struggling. Under such conditions, expecting charity to sustain a sufficient number of NMOs is unrealistic, which leaves Cagé’s proposal at odds with the desideratum of media pluralism.

This might change if public culture shifted and citizens began to prioritize journalism. In such a case, investment in NMOs would likely rise, increasing their number. Still, even then, the proposal would remain problematic from the standpoint of media pluralism, especially in a context of deep economic inequality. Even if most citizens chose to donate, their capacity to do so would be unequal: wealthier individuals could contribute more, leading to the NMOs aligned with their preferences being better funded. This would skew the civic media subsector toward the views of the upper classes, sidelining those of lower-income groups. This is not a very extravagant hypothesis, as that’s precisely what already happens in the extant philanthropy-funded media, where donations disproportionately come from the upper-middle classes and the (super)wealthy (Benson, 2018; Scott et al., 2019), who tend to fund news media they know, that align with their worldviews, and that are located near where they live (Usher, 2021: ch. 7). Thus, unless Cagé’s proposal introduces some redistribution of resources toward lower-income groups, it would probably fail to avoid the class biases so typical of philanthropy-funded media.^4^

Note that tax incentives are unlikely to change this dynamic. By making donations cheaper for all, they would disproportionately benefit the wealthy, who would save more. As noted, this saved money is tax revenue the state would no longer collect, and could *not* redistribute, then, to the worse off—for instance, through news vouchers that could empower them to support their preferred outlets. Thus, by subsidizing NMOs via tax breaks, Cagé’s plan would indirectly fund the media favored by the rich, and it would do so at the cost of undermining a more democratic media system by stripping the state of resources needed to build it.^5^

Note also that the unequal distribution of voting rights seems unable to counteract these class-based effects. In comparison to traditional philanthropy-funded outlets, NMOs would be less subject to the influence of large donors. But that is precisely why large donors would have also less incentives to invest in NMOs than in ordinary news media, where investment is proportionally corresponded with voting rights. As Schiffrin (2021: 8) notes, when the economic incentives to invest in a news media organization are low, “it becomes increasingly likely that rich individuals with a political agenda will seek ownership of the media outlets to further their agendas”.^6^ Now, if to the lack of financial return we also add the lack of influence, it seems that the only remaining motivation for large donors to fund NMOs would be altruism. And it seems to me overly optimistic to assume, as Cagé appears to do, that there will be enough altruistic donors to sustain a sufficiently large and diverse civic media subsector.

Yet, even if the wealthy were benevolent enough, two other problems would persist.

The first problem is that Cagé’s bylaw does not ensure that NMOs meet any standard of journalistic quality, so it cannot guarantee that NMOs will produce quality journalism. As currently conceived, the bylaw would enable almost any organization to qualify as an NMO and thus access the (indirect) public funding meant to fund journalism. While the nonproportional voting rights might foster internal democracy by mitigating power asymmetries, this alone cannot ensure NMOs will be minimally representative of diverse social groups, adequately professionally staffed, or even sufficiently independent from parties, lobbies, or religious associations. To be clear: I do not claim that all the organizations failing to meet these standards should be barred from public support—there may be reasons to fund them. My point is that we should not expect such entities to produce quality journalism, and that they should therefore not receive funds earmarked for that purpose.

The second problem is that, if the production of journalism depends on the goodwill of a few wealthy donors, then citizens’ right to receive quality news will not be robustly protected. For rights to be robustly protected their fulfillment should be guaranteed across different plausible hypothetical scenarios. That is why rights are often conceived as *“*trumps*”*: having a right to something means that you can enjoy whatever the right entitles you to, independently of contingent factors such as others’ preferences (Dworkin, 2009). Imagine, for example, the absurdity of a right to free speech that only allows you to speak if others agree with you. When applied to social rights such as the right to healthcare or to journalism, which entitle their right-holders to receive a good or service, this means that there must be a system that robustly provides that good or service regardless of others’ preferences.^7^

To make my case clearer, imagine a healthcare system where healthcare services are delivered by Nonprofit Health Organizations (NHOs) funded primarily by voluntary donations. In such a system, you may receive decent healthcare *if enough donors care about public health*. If not, you may receive poor or no care at all. Furthermore, if you have access to some healthcare, the treatments you could receive would reflect which conditions the donors worry about, as they would presumably fund NHOs specializing in treating those conditions. Thus, you might get treatment only if you are lucky and suffer a condition donors care about. This scenario is concerning because your right to healthcare would not be *robustly* protected: the fulfilment of your right to receive medical treatments would ultimately depend on others’ arbitrary whims. In a media system formed by NMOs, a similar problem would affect citizens’ right to journalism, as the fulfilment of this right would depend on whether sufficient donors are willing to fund quality journalism. These donors may not only condition *which* kind of journalistic service is available to citizens, which in itself would be problematic on pluralism grounds; they may also withdraw their support at any time, leaving citizens without access to *any* journalistic service *at all*.

To be sure, the incapacity of Cagé’s proposal to grant robust protections for the right to the news is not a silver bullet against it, but it shows that NMOs—and, more generally, philanthropy-funded news media—should ideally play only a secondary role in the fulfilment of citizens’ right to quality news: other mechanisms should robustly ensure that citizens are provided a regular supply of quality news *regardless of whether the rich are willing to fund that supply*. Notably, such a supply might not necessarily come only from public media and advertisement-funded private outlets, but also from a private *civic* media subsector robustly funded with public money.

## Latham’s Voter-Funded Media System

The second proposal is owed to Latham (see esp. 2007; 2012a; 2012b). His idea consists of distributing a fixed amount as cash prizes among competing informants based on the votes they receive. These informants, which may include bloggers, teams of journalists, or freelancers, are what Latham calls *voter-funded media* (henceforth VFM). The contest would be administered by an independent body, such as the non-profit *Votermedia*, to which Latham himself is associated, or a public institution (Latham, 2012b: 4, 7). Unlike other proposals, the VFM system has been tested on a small scale, although the idea is applicable “in democracies of any size” (Latham, 2007: 30).^8^

Latham’s proposal has three key traits. First, the system incorporates pretty permissive eligibility criteria—as I refer to the conditions media organizations must meet to compete for public funds. In the small-scale university VFM system, only two conditions were imposed. The first was a modest entry fee (between $100 and $150) meant to discourage unserious or unprofessional informants. The second required that informants were not candidates for, or members of, the student council whose elections they would cover—thus aiming at preserving VFM’s independence.

A possible way to adapt Latham’s idea to the large scale would thus be to escalate these criteria accordingly. For instance, the entry fee could be raised to match the higher financial capacity of potential contestants, thereby escalating its capacity to deter non-serious informants, while the incompatibility rule could be reformulated to exclude any media outlet that includes candidates or elected officials among its ranks. Alternatively, large-scale VFM systems might dispense with eligibility criteria altogether. This seems to be Latham’s preferred view, as he suggests that “the contest should be open to any individual, group or organization,” including amateur bloggers and professional journalists, whether independent or affiliated with a news outlet (Latham, 2012c: 4). As we’ll soon see, both options are problematic.

Second, VFM systems would involve two parallel competitions (Latham, 2012a: 5–6; 2007: 17–18). The main one is an *election-bound competition*: it would begin shortly before an electoral campaign and run concurrently with it. Prizes from this competition, which would distribute most of the available funds, would be awarded based on votes cast while voting for candidates—perhaps through a dedicated section on the ballot. The second, *continuous competition* would operate year-round, allowing voters to support their preferred media via an online platform. Regular tallies (e.g., weekly or monthly) would determine the distribution of smaller prizes. This two-tier system is designed to promote continuous media engagement while intensifying coverage around elections.

Third, the prizes would be calculated through a consensus-based method, whereby each contestant receives the median amount recommended by voters (Latham, 2012b: 5; 2007: 18).^9^ Unlike the *average*, which is calculated by summing all values and dividing by the number of votes, the *median* is the middle value in a list of values ordered from lowest to highest. If the number of values is odd, the median is the one at the center; if the number of values is even, then the median is the average of the two central values in the list. Thus, in the list “2, 3, 4, 5, 9”, the median is 4, while in the list “2, 4, 5, 9” the median is 4.5. Note that this allocation method requires that at least 50% of voters approve giving *some* funds to a media outlet for it to receive *any* money. To illustrate, consider a simple VFM system with 9 voters and 3 competing outlets. The table below shows a possible distribution of recommended prizes. According to Latham’s consensus-based method, each outlet receives the amount in the fifth position of its ordered list, so only VFM1 would receive a prize.

**Table:** 

VFM 1	1000	1000	800	800	700	500	200	0	0
VFM 2	1000	1000	1000	0	**0**	0	0	0	0
VFM 3	1000	500	300	200	**0**	0	0	0	0

The aim of this mechanism is to ensure that only “media that appeal to a broad cross-section of voters” can get prizes, thereby avoiding the risk that media serving “narrower interests or extreme views” receive any public money (Latham, 2012b: 5). This risk is, in Latham’s view, inherent to voucher systems such as Ackerman’s and McChesney and Nichols’, where each voter allocates “her own small slice of public revenue to whichever media she chooses” (Latham, 2016: 4). As we’ll see, thing are not exactly like that, as voucher systems do have criteria of eligibility that restrict which media opt to public funds. Latham is right, however, in noting that the existing voucher schemes are too permissive. His system thus appears well-designed to avoid some drawbacks that undermine other proposals—such as the risk that resources meant to support quality journalism are diverted to other activities, or the lack of funding, which severely affects Cagé’s proposal. And yet, as I will now argue, the VFM system remains far from perfect.

The first problem with Latham’s system is precisely its excessive permissiveness. Even if adapted, the eligibility criteria he proposes would not ensure that VFMs meet minimal institutional standards of quality journalism.

Consider first the entry fee. If kept low, it would fail to deter unserious participants and thus serve little purpose. But raising it would also be inefficient: high fees would not block unreliable sources—such as media outlets controlled by political parties, lobbies, or large corporations—as they could easily afford paying the fee. Meanwhile, smaller, less resourced, but not necessarily untrustworthy, organizations might struggle to enter the contest. Thus, by screening candidates based on their financial means rather than their professionalism, inclusiveness, or independence, the entry fee produces both false positives and false negatives.

Consider now the incompatibility criterion. While it might suffice in student council elections, where the stakes are presumably too low to create formal political factions, it is inadequate for elections with real political power at stake. Such contests attract wealthy, influential actors—such as parties, lobbies, or corporations—who could use VFMs to serve their interests. If the goal is to ensure that public funds support politically independent media, then merely requiring that VFMs exclude candidates or elected officials from their ranks is not enough. To illustrate: note that, under Latham’s reformulated criterion, media outlets owned and operated by political parties would still qualify. Ensuring VFMs’ independence requires, then, stronger criteria of incompatibility.

Even if adapted to the large scale, Latham’s proposed criteria are thus ill-equipped to select minimally competent and independent news media. Moreover, note that, if we followed his suggestion of abandoning eligibility requirements altogether, then the system would become even more permissive and therefore even less effective at filtering out low-quality candidates.

Admittedly, using Latham’s consensus-based method to fix the value of prizes might offset the excessive permissiveness that might result from his scheme’s eligibility criteria, or from the lack thereof. However, Latham’s consensus-based method is also problematic in its own right, as it seems to make it virtually impossible for a sufficiently large number of outlets to receive funding. Recall that, under Latham’s scheme, a media outlet must receive support from at least 50% of voters to receive *any* public funds. In large-scale settings, such consensus is highly improbable. In polarized societies, different factions may actively boycott each other’s preferred media, recommending they receive nothing. But even in non-polarized democracies, it is likely that most citizens allocate prizes to just a few, their preferred or best-known outlets.^10^ It is therefore reasonable to expect that media outlets attracting convergence from at least 50% of the citizenry would be extremely rare—if any exist at all. Notably, local and regional outlets would have virtually no chance of success in a national VFM system. Thus, while the consensus-based method would effectively block low-quality outlets—thereby compensating for overly permissive eligibility—it would do so at the cost of making it virtually impossible for most, if not *all*, organizations to get any actual funding. This is hardly compatible with the media pluralism desideratum.

A second problem with VFM systems stems from their *ex-post* funding structure. As seen, VFM systems would distribute most funds retroactively as prizes, rather than proactively in the form of investments or donations (Latham, 2012a: 4; 2016: 5).^11^ While this retrospective model may work at a small scale, where VFM could operate with modest resources, producing quality journalism for an entire polity is a far more demanding task requiring substantial investment—investment which small-scale entrepreneurs often cannot secure independently. As a result, in large-scale VFM systems, competitors would be forced to seek external funding upfront to launch and sustain operations until the main prizes are awarded.

This seems unacceptably classist: VFMs able to finance their operations through alternative sources, such as advertising or donations from wealthy donors, could enter the competition and participate with relative ease. Meanwhile, minoritarian organizations without the backing of affluent patrons would enter with fewer resources and likely under greater financial pressure, especially if they incur debt to participate. Compared to those already well-funded by external sources, these disadvantaged outlets would be more prone to underperformance. For instance, they would probably struggle to cover all relevant topics, or to do so in a timely manner. As a result, it is likely that most cash prizes would flow toward VFMs already well-funded, rather than to genuinely needy media. Thus, ex-post funding systems are prone to create a Matthew effect: those who already have more will likely end up receiving more, not due to merit or need, but to preexisting advantage.

It is also troubling that the very source of this competitive (dis)advantages may undermine the independence of VFMs. If those receiving external funding make a substantial portion of their income from wealthy donors or advertisers, they would be disincentivized from scrutinizing or criticizing their patrons, out of fear of losing a significant share of that income. At the same time, if smaller VFMs incur debt to participate in the competition, their independence may be similarly constrained, as they would have strong incentives to avoid criticizing their creditors—at least until those debts are repaid. Perhaps winning several prizes could eventually allow smaller VFMs to regain full editorial autonomy, but until then, what was intended as a system of publicly funded media would look more like a competition among privately funded outlets vying for publicly subsidized bailouts.

## Ackerman’s Internet News Voucher

The third proposal, advocated by Ackerman (2011a, b; see also Ackerman, 2013* internet news voucher*. The idea is to distribute a public budget line among various digital news outlets in proportion to the number of votes their individual articles receive. The mechanism would be simple: when readers find that an article has enhanced their understanding of public issues, they should be able to click a button on the article’s webpage. Each click would count as a vote, and these votes would then be translated into monetary vouchers, which the *National Endowment for Journalism* would distribute to the creators of the content.

Note that, in Ackerman’s scheme, the voucher refers to the amount of public money distributed to each media outlet, which is calculated through “a strict mathematical formula: the more clicks, the bigger the check from the Endowment.” (Ackerman, 2011b: 43). Note also that the system allows citizens “to click in support of particular articles” (2011b: 45), rather than entire organizations. This design, he argues, is better suited to the digital era, where readers typically consume journalism through aggregators rather than directly on news outlets’ websites. Being more “user-friendly,” this click-based model is “likely to generate far broader participation” (2011b: 45) than systems such as Latham’s or McChesney and Nichols’, which require a more formal voting process. Additionally, since the system allows citizens to vote at any time—rather than during a single annual event—it fosters “a more modulated and ongoing citizenship response” (2011b: 45). This continuous feedback—similar to that generated through the VFM-system’s *continuous competition*—enables media organizations to more accurately gauge public appreciation and adjust their work accordingly.

Ackerman’s scheme includes two eligibility criteria. The first excludes pornographic content, which should not be funded “even if some of its viewers cynically check the box asserting that it has ‘contributed to their understanding of public issues’” (Ackerman, 2011a: 303). The second requires that eligible media outlets pass a “market test” (2011a: 303): they should get “an insurance policy to cover the costs of compensating people whose reputations they destroy through false reporting”, which would require them to “go into the marketplace and satisfy an insurance company that they have the resources to do serious fact-checking.” (2011a: 303). Although Ackerman claims that the immediate purpose of this insurance requirement is to prevent public funds from “subsidizing libel” (ibid.), we might read it more broadly as an attempt to prevent low-quality outlets from competing for public funding.

To complement these criteria, Ackerman proposes two anti-scam measures designed mostly to prevent bot voting and voting by paid “professional clickers” (2011b: 46). First, a basic Turing test: before clicking, users would be required to spend a few seconds typing “random words or syllables” (2011b: 44). Second, the system should “accept only a single click from any computer for an article” (2011b: 46 fn. 3). Beyond these general restrictions, Ackerman argues that “we should leave funding decisions to the countless clicks of ordinary citizens” (2011b: 44). His system is thus highly permissive: virtually any individual or organization could compete for public funds.

My first criticism is precisely that Ackerman’s scheme is *too* permissive. So much so that it allows news media that fail to meet even minimal standards of quality journalism to compete for, and receive, public funds. This concern is not only mine. Ackerman himself concedes that “[t]here will be lots of clicks for scandal and mongering and the like” (2011b: 45). Yet, rather than proposing more stringent eligibility criteria, he bites the bullet and says that:

“that’s the price we have to pay for a system that will also generate serious journalism: it’s one thing for the government to screen out pornography and punish libel; quite another, for it to claim the power to distinguish between “serious” and “sensationalistic” news accounts.” (Ackerman, 2011b: 45 − 6).

In my view, however, we do not need to pay such a high price to fund “serious” journalism. Of course, under non-ideal circumstances, some spillover is inevitable: a portion of public funds intended to support quality journalism may end up diverted elsewhere. But we need not accept so comfortably that a large share of funds will be misallocated. Instead, we should aim to minimize the proportion of public money diverted toward outlets that are not institutionally equipped to produce quality journalism. And, contrary to Ackerman’s suggestion, such efforts do not require that “the government” itself distinguish between “serious” and “sensationalistic” content.

For starters, civic media policies need not—and indeed *should* not—grant the government the power to decide where the money goes (McChesney & Nichols, 2010: 204). A more desirable alternative is to entrust an independent institution with the task of verifying, on impartial and nonpartisan grounds, which actors meet the eligibility criteria. This approach aligns with the proposals of McChesney and Nichols, as well as of Ackerman himself, which envision the creation of an autonomous body—respectively, the *Citizenship Media Fund* and the *National Endowment for Journalism*—to manage and oversee the civic media system (cf. Rolnik et al., 2019: 36, 46). To be sure, the risk of political capture is real. But this risk is not specific to civic media oversight bodies; it applies to any public institution expected to operate independently. The concern that such a body would inevitably be subordinated to the government is therefore no more justified than similar concerns about the independence of central banks or constitutional courts. If we were able to design central banks and constitutional courts that operate reasonably well at an arm’s length from the government, there is no reason to believe we could not do the same with a hypothetical *Civic Media Fund*.^12^

Besides, unlike Ackerman seems to assume, civic media policies need not—and, indeed, for the most part, *should* not—discriminate which *content* may be financed. Instead, civic media policies should aim to establish which *organizations* are trustworthy enough to be allowed to compete for public funding. If in the past an organization has produced content clearly incompatible with the journalistic function—such as pornography, hate speech, or fake news—or if there is well-founded reason to believe that it would use public funds to produce such content, then it may justifiably be deemed ineligible. However, except in such extreme and clear-cut cases, civic media policies should refrain from evaluating *content*. Instead, they should determine eligibility primarily by examining whether media *organizations* exhibit institutional traits that show they are reasonably trustworthy to produce high-quality journalism, enabling those who exhibit those features to compete for public funding, and barring those who fail to minimally exhibit them.

Of course, agreeing on which are the relevant traits and which institutional characteristics exhibit those relevant traits are difficult tasks, arguably the most difficult ones when it comes to designing civic media policies (cf. Kurtulmuç and Kandiyali 2023: 284; Starkman & Chittum, 2021: 254).^13^ Whichever these traits and institutional characteristics are, it seems nevertheless clear that there is room for more demanding aspirations than those Ackerman indulges himself to harbor—without necessarily granting the government control over the news. We might, for instance, require that eligible media are not formally affiliated with, or funded by, any political party—something that, contrary to Ackerman’s apparent concerns, would probably *prevent*, rather than promote, government influence over the media. Probably, even with such eligibility requirements, some public funds intended for quality journalism may still be diverted. However, the volume of diverted funds would likely be much smaller, and thus the price paid for a system that delivers “good journalism” would be significantly lower.

The second major concern about Ackerman’s proposal is that it entrusts the market—specifically, insurance companies—with the task of screening out low-quality news media, thereby opening the gate to various pernicious distortions. Some of these distortions stem from insurance companies leveraging their position to advance their own interests. It seems naïve to believe that insurance companies would base their decisions solely on economic factors—which would be already problematic on media pluralism grounds. Being realistic, we can expect them to also consider ideological affinities, for example, giving preferential treatment to outlets that support the privatization of public services like healthcare—since such privatization would expand their market share as insurance providers—while refraining from insuring outlets that oppose privatization and advocate for universal, free, and public healthcare. More generally, media with libertarian or pro-capitalist agendas would probably have an easier time obtaining insurance than those with socialist or welfarist views.

One might argue that insurance companies should be required to offer coverage to all news media according to objective standards, so that if a media organization meets those conditions, insurers would not be allowed to deny them coverage. Perhaps this is what Ackerman has in mind when he suggests that there will need to be “some governmental monitoring of insurance companies” (Ackerman, 2011a: 305). But this cannot be a solution, for it merely begs the question of which conditions media outlets must meet for insurance companies to be obligated to insure them. Those conditions are, precisely, what eligibility criteria are supposed to define in the first place. So we are back to square one.

An additional concern about the insurance requirement is that it may encourage strategic litigation. Those seeking to undermine a media outlet could use lawfare-like tactics, filing defamation cases to damage its reputation, reduce insurers’ willingness to provide coverage, and potentially force the outlet to pay higher premiums. We need not be very imaginative to see this kind of strategic litigation happening. As Schiffrin (2021: 8–9) notes, “[t]he use of lawsuits to shut down journalism is now common in many parts of the world. It’s part of a strategy of intimidating journalists”. Ackerman’s insurance requirement is thus problematic, obviously, because it would entail an extra burden for the media targeted by these perverse forms of litigation. But note that in Ackerman’s scheme even media not directly targeted could be very easily intimidated, and have incentives to avoid critical reporting on issues or individuals likely to trigger defamation claims. Notably, the incentives to offer timid reports—or to refrain from reporting altogether—increase with the power of the individuals who might be criticized. Worryingly enough, this anticipates that, under Ackerman’s scheme, eligible media would be incentivized to act as an *inverted watchdog*, one that is lenient with the powerful and harsh with the powerless. This would be at odds not only with the deliberative, but with virtually *any* ideal of journalism (see Christians et al., 2009: ch. 6).

My third criticism is that, by not limiting the number of clicks each citizen can cast, Ackerman’s proposal would allow some individuals to vote more than others, granting them disproportionate influence over funding decisions and, by extension, over the whole civic media subsector. This is concerning because those with less free time and resources—typically members of disadvantaged groups (see Herzog, 2024)—are likely to cast fewer votes, which would in turn introduce a class bias into the allocation of funds. Similarly, highly radicalized citizens may vote more, as they might be willing to pass the Turing test on multiple devices, while more civic-minded individuals probably would not. This would add a partisan bias to the distribution of funds. That bias could be even further amplified by partisan outlets, which might harvest more clicks by publishing numerous short articles for their devoted audiences. Indeed, not only partisan outlets, but all the eligible media would have incentives to produce short, and therefore superficial, pieces rather than longer, in-depth analysis, simply to maximize click counts. All this points to the need for policies that generate proper incentives—both for citizens and eligible media—and distribute fairly among citizens the power to allocate funds.

A fourth and final problem is that the internet news voucher system favors certain types of media organizations—such as digital newspapers and magazines—which, due to their format, typically make it easier for audiences to express support for their contents through ‘clicks’. In contrast, other media organizations—such as those producing radio or television—offer the news in formats that make it significantly more difficult to register support, especially if support must be expressed for individual news items. Of course, these organizations could maintain websites where citizens might ‘click’ for their contents, but doing so would involve an additional burden—with a subsequent comparative disadvantage that would hamper format pluralism. A plausible way to address this imbalance is to establish a universally accessible digital platform, independent of all news outlets, where citizens could express their preferences regarding the allocation of public funds for journalism.

## McChesney & Nichols’ Citizenship News Voucher

The last proposal, put forward by McChesney and Nichols (2010: 201–06; see also McChesney, 2013: 211–14; 2016), is the *citizenship news voucher*. In a nutshell, the idea is that each citizen would receive an annual, publicly funded voucher—proposed at a value of $200—which they could donate to non-profit news media of their choice.^14^

Note that, unlike in Ackerman’s scheme, here the term ‘voucher’ refers to the fixed amount of money granted by the state to each citizen, rather than to the variable amount transferred to each news outlet. Citizens could divide the amount between different eligible outlets or choose not to spend all (or any) of it. Donations would be made through a formal procedure, which McChesney and Nichols suggest integrating into the tax return process (those who do not file tax returns could complete an alternative form). Funds would then be transferred to media organizations according to citizens’ expressed preferences. Any unspent funds would return to the *Citizenship Media Fund*, a public agency independent of the government, tasked with two main functions: determining which outlets meet the eligibility criteria and distributing funds among them according to citizens’ choices.

McChesney and Nichols (2010: 202) propose five criteria of eligibility. First, eligible news media “cannot be part of a larger organization”; instead, they must be independent entities. Second, they must be non-profit associations, which means they should distribute no dividends and reinvest all profits into their activities. Third, they must “do exclusively media content”, thus avoiding engagement in “any non-media operations” (McChesney & Nichols, 2010: 202) such as, for instance, music festivals or pornography. Fourth, eligible media must not finance themselves through advertising, as in their view “this is a sector that is to have a direct and primary relationship with its audience” (2010: 202). Interestingly, though, McChesney and Nichols’s scheme would allow eligible media to “accept tax-deductible donations from individuals or foundations to supplement their income” (2010: 202). Fifth, content funded through vouchers should not be copyrighted and should be made freely and immediately available online. For McChesney and Nichols (2010: 202), this is a matter of justice: if content is publicly funded, it should also be publicly accessible (see also McChesney, 2013: 212; 2016: 134, 136).

To these five criteria, McChesney and Nichols add what I call the *minimum threshold proviso*: for any eligible media outlet to effectively receive funds, the proposed donations in its favor must reach at least $20,000. This threshold serves two purposes. First, it ensures that funded media are financially viable, at least for a few years (McChesney & Nichols, 2010: 204). Second, it prevents scams by making it impossible for an individual or small group to donate their voucher(s) to themselves, as meeting the $20,000 threshold would require at least 100 people banding together (McChesney & Nichols, 2010: 203).

All these requirements establish a more demanding framework than those outlined in previous proposals, which reflects McChesney and Nichols’ greater concerns both with preventing corruption and with ensuring that outlets receiving public funds meet minimum standards of quality. And yet, despite being arguably the most sophisticated scheme, the citizenship news voucher system also has shortcomings.

The first foreseeable problem is the risk of *pre-allocation media campaigns*. Since the distribution of funds is decided at a specific moment, we may expect that just before the allocation period eligible media strive to provide better service, spending more and making greater efforts to gain support, engaging also in harsher—though not necessarily constructive—criticism of competing media. Once the allocation is decided, eligible media would have incentives to save resources for the next campaign, thereby offering a poorer informational service in the meantime. Lump-sum allocation systems are thus problematic because they incentivize outlets to provide irregular coverage throughout the year based on funding schedules rather than on journalistic considerations about what citizens need to know. Once again, this fear is well-grounded. As Starkman and Chittum (2021: 248) report, in Hungary—where citizens can choose to donate 1% of their personal income tax to organizations of their choice—“[e]very spring before the tax filing, public-benefit organizations (…) conduct marketing campaigns to convince taxpayers to designate their 1% to them.” In these campaigns, media organizations that opt for this form of funding strive to persuade people to allocate their 1% to them, rather than to any other association—which in this scheme includes not only competing media outlets, but also charitable organizations, the Central European University, and the Catholic Church. To avoid such monetary incentives from conditioning the functioning of the media, it seems preferable to implement a continuous allocation system that allows citizens to donate their contributions at any time, thereby creating incentives to maintain a high-quality informational service year-round.

One might argue that fund allocation should remain a one-off payment but timed to coincide with elections—as in Latham’s election-bound competition. Since elections are presumably when citizens need more information, the incentives created by pre-allocation media campaigns would then align with citizens’ heightened informational needs.^15^ I believe, however, that lump-sum allocation systems—even if aligned with elections—are less desirable than continuous allocation systems. First, it is doubtful that citizens need only minimal information outside electoral periods. After all, public affairs are too complex to be understood in the short run-up to voting. Besides, citizens are expected not just to vote once every few years, but also to engage in year-round deliberation, oversight the government, and—if needed—contest unjust policies.^16^ Hence the need for a good supply of news throughout the year. Second, election campaigns tend to polarize society. Tying media funding to elections risks importing this polarization into the civic media subsector, as citizens’ donations during campaign periods may be more affected by partisan loyalties. A continuous allocation system avoids both problems by decoupling fund distribution from the electoral calendar. It incentivizes eligible media to maintain high-quality coverage year-round, while giving them the autonomy to intensify efforts whenever elections, or any other newsworthy events, occur. Crucially, during such periods of enhanced coverage, outlets would face fewer incentives to unnecessarily attack their competitors, not only because there would be no fixed allocation moment coinciding with those periods, but also because different outlets might choose to intensify coverage at different times.

A second problem is that McChesney and Nichols do not specify what would happen to funds donated to media outlets that fail to meet the threshold of donations, and no option seems straightforward.

One option is returning non-transferred funds to public coffers, along with the money citizens decide not to allocate to any media. But this would be unfair to those who tried to support media outlets that fell short of the threshold, as it would effectively nullify their influence over funds distribution. More troublingly, it would disadvantage minoritarian outlets, which would have more difficulties in attracting voucher money: knowing that donations might not be executed unless the recipient reaches the threshold, citizens would be incentivized to favor safer, more popular outlets.

A second option is freezing the non-transferred donations until the next allocation process, executing them if the threshold is then met. This, too, raises several problems. First, in some cases it would be implausible to expect that a donor’s contribution will ever become effective: if you donate your $200 to an outlet that nobody else supports, and assuming a $20,000 threshold, your first donation won’t become effective unless you donate all your voucher money for the next 99 years. While extreme, this example illustrates how supporters of minoritarian media could easily find their funds locked for many years, or even lost, until their chosen outlet becomes operational. In the meantime, their influence over funds distribution would be lower than that of other citizens. A second problem is that, if donations remain frozen, scams would be easier: it would no longer be necessary for 100 people to band together to funnel voucher money to themselves; 50 people with 2 years of patience would suffice, or just 25 people if they wait 4 years.

A third option to fix the problems with the threshold would be to cancel non-transferred donations and increase the donor’s next voucher proportionally. Thus, for instance, if you donate $50 of your voucher dollars to an outlet that fails to reach the minimum donation threshold, your voucher next year will be worth $250 instead of $200. This option seems preferable in that, by allowing citizens to reallocate their funds later, it preserves their influence over funds distribution. Still, it remains imperfect, as coordinated groups could again easily scam the system by accumulating non-transferred funds and then donating their inflated vouchers to themselves. Besides, one might wonder: why should citizens have to wait a whole year to redirect their non-transferred donations? Why shouldn’t they be able to redirect their donations at any time? All this suggests that, if a threshold of minimum donations were implemented—as seems reasonable on the grounds McChesney and Nichols identify—, we would have additional reasons to introduce continuous allocation mechanisms that enable citizens to order and modify their donations at any time.

The third problem is that McChesney and Nichols focus primarily on ensuring that eligible media outlets are economically independent, which leads them to neglect the equally important need of *political* independence.^17^ Note that, of their five criteria, the only one aimed at promoting political independence is the prohibition that eligible media belong to any other association. This restriction rightly includes political parties, lobbying groups, and religious organizations, which are barred from owning eligible media outlets. However, the fact that one association is not *formally* part of another does not guarantee the absence of dependency between them. An outlet may comply with the formal independence requirement while still being *de facto* controlled by another organization. For instance, all members of an eligible outlet might simultaneously belong to—or even hold leadership positions within—a political party, lobby, or religious group. Thus, even if the outlet is not officially owned by, or structurally integrated into, any such entity, it is reasonable to expect that its editorial decisions could be shaped more by the interests of that party, lobby, or religious group, than by journalistic considerations. The lack of eligibility criteria tackling these links is remarkable not just because it undermines an abstract aspiration to media independence, but also because McChesney and Nichols (2010: 161) explicitly stated their aspiration to preserve the “Chinese wall” between politicians and media. Future proposals should therefore include more stringent and better tailored criteria to ensure that eligible outlets are not only *formally* independent, but autonomous enough to base their editorial decisions mostly—if not *only*—on journalistic considerations about what citizens need to know.

The fourth problem is that, despite McChesney and Nichols’ emphasis on economic independence, their proposed eligibility criteria seem also insufficient to ensure that eligible media outlets will be genuinely independent from an economic standpoint. To be sure, some of their requirements—especially the non-profit and no-advertising criteria—may successfully prevent the for-profit logic of the market from infiltrating newsrooms. However, economic dependence can arise from sources other than the market. In this case, economic dependence is likely to come from private donations. Recall that in McChesney and Nichols’ scheme eligible outlets could receive *unlimited* private contributions. We may expect, then, that some outlets will seek supplementary funds from private donors. Given the interests at stake in the media system, and considering the socio-economic inequalities of our societies, we may reasonably expect the main private donors to be (super)wealthy individuals, large corporations, lobbies, and political parties. This is concerning, for—as noted in Sec. 2—once the economic prospects of a media organization substantially depend on the good will of a few donors, we may expect it to give disproportionate consideration to the interests of those donors. When that happens, the organization’s editorial independence is compromised.

Of course, civic media could refuse such donations, and some surely would. But as long as unlimited private contributions are permitted, some outlets will surely accept them. Once they do, they will be better positioned to compete for voucher funds, placing donor-rejecting outlets at a disadvantage in the competition for public money. This dynamic may gradually push even the most principled outlets into accepting (more) private donations, thereby compromising their independence too. I don’t mean to suggest that private donations should be categorically prohibited. Philanthropy can indeed have positive effects, such as providing resources that would otherwise be unavailable (see Ceva, 2021). My point is, rather, that allowing unlimited private donations risks distorting the playing field in favor of the outlets more willing to compromise their independence, generating a structural drift toward increasing reliance on potentially compromising sources of funding. Harnessing the benefits of philanthropic support for journalism, while minimizing its dangers, requires, then, a more fine-grained regulatory framework than the one McChesney and Nichols propose.

The fifth and final problem is that McChesney and Nichols’ proposal cannot ensure that eligible media outlets meet minimal standards of journalistic quality. Despite incorporating more demanding eligibility criteria than other proposals, their scheme does not require organizations to be even minimally professional, inclusive, or—as just shown—independent. As a result, their system could not prevent public funds from being diverted toward outlets producing extremely low-quality content. Indeed, McChesney (2016: 134) himself concedes that the proposal could easily end up funding “fascist newspapers”. And yet, just as Ackerman, McChesney and Nichols (see esp. 2010: 205-6) seem resigned to accepting this as the price to pay for a more democratic media system.

As said, I believe this is a bullet no civic media policy should bite. If the purpose of civic media policies is to fund quality journalism with public money, then they should aim to ensure that as much of that public money as possible is effectively invested in good journalism. If in non-ideal circumstances that is not fully possible, then they should at least aim to prevent public money from funding extremely low-quality content, or goods entirely unrelated to the policy’s purpose. Abandoning this aspiration, and accepting that eligibility criteria can be so lax as to permit the funding of virtually anything, undermines the whole purpose of civic media policies.

## Conclusion: Lessons for Future Civic Media Policies

The previous assessment reveals major challenges for civic media policies to fulfil their promises. One might be tempted to conclude that the problems affecting these proposals justify dismissing the civic strategy for good. That would be too hasty a conclusion, though. We should bear in mind that the problems affecting extant proposals cannot justify rejecting the whole strategy altogether, just as the flaws of some public education policies cannot justify rejecting public education altogether. Besides, whether a specific civic media policy can fulfil its promises depends *inter alia* on contextual factors that vary across societies. The same policy could work, then, in one country and be a disaster in another. It is thus possible that after careful consideration of all the relevant factors, we conclude that there are not sufficient reasons to try any civic media policy at all.^18^ Having said that, I hasten to note that here I have assessed only four proposals, which obviously cannot exhaust the list of possible civic media policies. There might be other, more promising proposals out there and—even if that’s not the case—better proposals might be conceived in the future. That is, indeed, my hope. As I see things, rather than a *tout court* rejection of the civic media strategy, the challenges identified throughout this assessment shed light on how to envision future proposals that are more capable of stimulating the proliferation of high-quality journalistic outlets. 

As advanced, the purpose of this article was not to craft any new proposal, but, more modestly, to draw some lessons about what those future proposals should look like. To that end, let’s recap the three specific promises implicit in civic media policies, and what the preceding analysis has revealed about each of them.

By allowing citizens to decide how public funds are distributed, civic media policies promise to be more directly democratic than their alternatives. But, as seen, some proposals fall short in ensuring that the power to influence funds allocation is fairly distributed. In Cagé’s system—as, more generally, in philanthropy-funded journalism—this power is shaped by preexisting wealth inequalities, while in Ackerman’s scheme it may vary depending on citizens’ free time or personal motivation. Latham’s and McChesney and Nichols’ proposals seem to fare better in this regard, as both guarantee that each citizen has equal influence by granting either equal votes or equally valuable news vouchers. Future civic media proposals should thus secure sufficient funds to guarantee that enough civic media exist, and make sure that the capacity to affect the distribution of those funds is fairly distributed among citizens. One of the key questions to be addressed here is whether the currency to be distributed fairly should be voucher money, votes, or something else. Moreover, it remains to be seen whether the distribution of this currency, whatever it may be, should be strictly egalitarian or follow other principle of justice, such as sufficiency or a Rawlsian Maximin-like one.

Civic media policies also promise to create a diverse media system and, more specifically, a diverse civic media subsector. However, some proposals fail to meet the desideratum of media pluralism even in its least demanding dimension—namely, numerical pluralism—as they cannot guarantee the proliferation of sufficient civic media. In Latham’s system, this is due to the consensus-based method for distributing funds, while in Cagé’s scheme the problem lies in the lack of warranted funding. That very same lack of warranted funds limits also Cagé’s scheme’s capacity to honour viewpoint pluralism, as her system would probably funnel most funds toward outlets catering to upper classes, who can afford to make philanthropic donations. Ackerman’s scheme, on its part, falls short of both viewpoint pluralism—due to its reliance on insurance companies to establish which outlets are eligible—and format pluralism—as its exclusive focus on digital media renders it unlikely to fund non-digital formats. The lesson here is that future civic media proposals should aim not only to ensure that sufficient funds exist but also strive to diversify their distribution among outlets that differ both in viewpoint and format. This, and particularly the need to achieve a minimum viewpoint pluralism within the civic media subsector, reinforces the importance of distributing fairly among citizens the power to influence funding allocation. Format pluralism, for its part, could be promoted by stipulating, for example, that citizens may not allocate all their publicly funded donations to a single outlet; instead, they might be required to divide the money among several eligible media, perhaps even among media that differ in some relevant aspect, such as format, area of coverage, or periodicity.

Arguably, the most important promise of civic media policies is that of funding independent, high-quality journalism. Unfortunately, this is precisely where all proposals perform the worst, as they all make it too likely that funds meant for quality journalism end up funding other things, such as entertainment or propaganda. Part of the explanation of this shortfall lies in the perverse incentives that the proposed schemes would generate if implemented. As seen when discussing Latham’s and McChesney and Nichols’ proposals, one-off allocation mechanisms incentivize media outlets to engage in pre-allocation campaigns, while ex-post funding mechanisms create undesirable Matthew effects. Besides, some schemes could incentivize citizens to scam the system, either by channeling funds to themselves—as it seems possible in McChesney & Nichols’ scheme—or by compulsively voting for their favorite outlets—as Ackerman’s proposal permits. To avoid these problems, future civic media policies should more carefully consider the incentives they generate, not only for eligible media outlets but also for citizens.

However, probably the main reason why existing proposals fail to ensure that public funds effectively support quality journalism lies in their inadequate eligibility criteria. Being excessively permissive, or even nonexistent, these criteria risk generating false positives, that is, allowing organizations that clearly fall short of even minimal standards of journalistic quality to compete for public funds. Simultaneously, the proposed eligibility criteria are likely to generate false negatives too, by disqualifying organizations that do meet such standards on seemingly arbitrary grounds. This is evident in Latham’s entry fee requirement, as well as in Ackerman’s reliance on insurance companies to determine eligibility. Thus, probably the most important lesson to be drawn from this analysis is that, notwithstanding the democratic potential of civic media policies, for them to deliver their intended outcomes, they should *not* be as *directly* democratic as one might initially wish them to be. This is so because, as seen, allowing citizens to transfer funds to any media organization would make it too likely that the funds intended for journalism end up financing something else. To prevent this from happening, future proposals should therefore incorporate better-tailored eligibility criteria. These criteria should serve as a filter that excludes some organizations while enabling others to compete for funding *on the grounds of their capacity to produce quality journalism.*

A detailed discussion of which criteria these should be goes beyond the scope of this article, but, in light of the preceding analysis, we can identify several good candidates. One such criterion is the requirement of being a non-profit organization, an institutional feature that offers a strong guarantee that the outlet will not be driven by market incentives. Another could be the requirement that eligible media outlet have a minimally professional and trained staff, as well as members representing the different relevant sociodemographic segments of society. Yet another possible criterion would be institutional autonomy, which should be defined not merely in formal terms—as in McChesney and Nichols’ framework—but more ambitiously, in material terms, as the absence of dependency relations that could unduly shape editorial decisions by making them responsive to non-journalistic considerations. Of course, these are just a few possibilities. Determining which criteria should ultimately be implemented, as well as how they should be specifically defined, is a terribly complex task—so much so that it might be considered “[t]he biggest challenge” (Starkman & Chittum, 2021: 254) for any civic media policy. Much more research and thought are needed to answer this, as well as all the other major pending questions about the civic strategy in media policy. I wish to believe that the preceding discussion has helped, even if just slightly, to pave the way toward answering them.

## Data Availability

Not applicable.

## Footnotes

1 But not only. Even if a particular civic media policy is well-suited to promote the proliferation of quality journalism, it might still be unjustified. For besides the *effectiveness* considerations on which I focus here, the full justification of a civic media policy should also take into account at least two additional kinds of considerations. The first are considerations of *distributive justice*, which concern whether it would be acceptable to raise—presumably through taxation—the resources required to implement such a policy. The second are considerations of *efficiency*, which concern whether the civic policy in question would be more cost-effective than the available alternatives.

2 For a discussion of different kinds of media pluralism, see Karppinen (2013).

3 Given the hybrid nature of NMOs, transferring money to them lies somewhere between investing and donating. Indeed, Cagé uses terms interchangeably, referring also to donors, investors, and shareholders indistinctly. I follow the same usage throughout this section.

4 Notably, Cagé (2020) has elsewhere extensively documented that donations to political parties are a “class phenomenon” (xiv), as “a much larger proportion of the superrich make contributions and, when they do, these are much larger than those of citizens on low incomes” (xix). It is surprising, then, that in the case of media she overlooks a similar phenomenon.

5 On how promoting philanthropy might shortcut the solutions to problems it aims to fix, see Ceva (2021). More generally, on the undemocratic nature of philanthropy, see Debief (2025).

6 Such is the case, as Schiffrin (2021: 8) herself observes, of Jeff Bezos, who has recently purchased *The Washington Post*, and Sheldon Adelson, who bought Israeli newspapers to support Prime Minister Benjamin Netanyahu. On this latter case, see also Grossman et al. (2022).

7 On the right to receive quality news as a social right, see Marciel (2023: 374-380). More generally, on social rights as positive rights, see Alexy (2002: ch. 9).

8 Indeed, the VFM system was originally conceived for—but never implemented in—the business world. To date, the largest experiment with voter-funded media has taken place during the annual elections of the Alma Mater Society, an association of 45,000 students at the University of British Columbia (Latham, 2012a). VFM case studies can be found at http://votermedia.org/. For the story of Latham’s VFM, see also Christopher (2012).

9 See also https://votermedia.org/wp-content/uploads/ubc/InterpolatedConsensus.html.

10 For evidence supporting this hypothesis, see Usher (2021).

11 Browne (2010) draws a similar distinction between ‘prior funding’ and ‘post facto reward’.

12 For insights into how that institution might be designed, and in addition to the works of Ackerman, Nichols, and McChesney, see, e.g., Cagé (2021), Hind (2010), Rolnik et al. (2019), or Sullivan (2021).

13 Although, as suggested in the introduction, at least from a deliberative standpoint three of such key traits might be professionalism, inclusiveness, and independence. See also the conclusion.

14 A very similar proposal, which instead advocated for vouchers worth $50, was later defended by a group of economists. Remarkably, that group also included Cagé (see Rolnik et al., 2019: 34–38).

15 This seems, indeed, Latham’s rationale for proposing that VFM systems distribute most funds through the election-bound competition (see Latham, 2012a: 5–6).

16 This is so not only for deliberative theory of democracy, but for most theories of democracy except, perhaps, the elitist view. For a comparison of different theories of democracy and citizenship, see Marciel (forthcoming).

17 Indeed, McChesney and Nichols (2010: 203) state that their voucher system has “two objectives,” both explicitly economic: first, to “dramatically increase their [i.e., of media organizations’] funding” and, second, to help them “become self-sufficient and even have the funds to hire a significant number of full-time paid workers”.

18 See also footnote 1.
